# Neonatal Intensive Care in a Karen Refugee Camp: A 4 Year Descriptive Study

**DOI:** 10.1371/journal.pone.0072721

**Published:** 2013-08-22

**Authors:** Claudia Turner, Verena Carrara, Naw Aye Mya Thein, Naw Chit Mo Mo Win, Paul Turner, Germana Bancone, Nicholas J. White, Rose McGready, François Nosten

**Affiliations:** 1 Shoklo Malaria Research Unit, Mae Sot, Thailand; 2 Mahidol-Oxford Tropical Medicine Research Unit, Bangkok, Thailand; 3 Centre for Tropical Medicine, University of Oxford, Oxford, United Kingdom; University British Columbia, Canada

## Abstract

**Background:**

A third of all deaths in children aged <5 years occur in the neonatal period. Neonatal intensive care is often considered too complex and expensive to be implemented in resource poor settings. Consequently the reductions that have been made in infant mortality in the poorest countries have not been made in the neonatal period. This manuscript describes the activities surrounding the introduction of special care baby unit (SCBU) in a refugee setting and the resulting population impact.

**Methods:**

A SCBU was developed in Maela refugee camp on the Thailand-Myanmar border. This unit comprised of a dedicated area, basic equipment, drugs and staff training. Training was built around neonatal guidelines, comprising six clinical steps: recognition, resuscitation, examination, supportive medical care, specialised medical care, and counselling of parents with sick newborns.

**Results:**

From January 2008 until December 2011, 952 infants were admitted to SCBU. The main admission diagnoses were early onset neonatal sepsis, jaundice and prematurity. Early prematurity (<34 weeks) carried the highest risk of mortality (OR 9.5, 95% CI 5.4–16.5, p<0.001). There was a significant decrease in mortality from 19.3% (2008) to 4.8% (2011) among the infants admitted for prematurity (p=0.03). The neonatal mortality in Maela camp as a whole declined by 51% from 21.8 to 10.7 deaths per 1000 live births over the corresponding period (p=0.04). Staff expressed more confidence in their ability to take care of neonates and there was a more positive attitude towards premature infants.

**Conclusion:**

Neonatal mortality can be reduced in a resource poor setting by introduction of a simple low cost unit specialising in care of sick neonates and run by local health workers following adequate training. Training in recognition and provision of simple interventions at a high standard can increase staff confidence and reduce fatalistic attitudes towards premature neonates.

## Introduction

Each year over three million neonates die. The commonest causes of death are due to the complications of preterm delivery, complications arising at the time of birth and from neonatal sepsis [1]. The majority of deaths occur in the first week of life and occur in the developing world [2,3]. As 2015 approaches, strides have been made in working toward the fourth millennium development goal, the two thirds reduction in childhood mortality [4]. However this reduction has occurred mostly in children outside of the neonatal period [5].

Intensive neonatal care in resource constrained settings, is often considered too difficult and too expensive [6]. However, effective neonatal care of very sick babies does not need to be highly technical and beneficial effects from training local health workers to provide skilled care has been reported [7].

Good neonatal care begins before birth with good antenatal care: improving nutrition in the mother, treating infections and providing tetanus immunisation [8]. A gold standard model of neonatal care combines good antenatal care, safe delivery, close proximity of obstetric and neonatal units and staff capable of providing medical care to the newborn infant.

Camps for approximately 160,000 refugees from Myanmar, mostly of Karen ethnicity, are spread along Thailand’s western border. Shoklo Malaria Research Unit (SMRU) is based in Mae Sot, Thailand, Northwest Thailand has been providing obstetric and maternity care to migrant and refugee populations along the Thailand-Myanmar border since 1986. Between 1986 and 2010, SMRU documented a six fold reduction in maternal deaths in women following their antenatal clinics in refugee camps on the border [9]. In addition, infant mortality decreased from 183 per 1,000 live births in 1987 to 78 per 1,000 live births in 1996 as a result of improved detection and treatment of cases of infantile beri beri [10]. The reported neonatal mortality rate in 2012 for Thailand was 8 per 1,000 live births and for Myanmar 32 per 1,000 live births [11,12].

The population impact of the introduction of neonatal care in a refugee camp from 2008 until 2011 is reported here.

## Methods

Maela camp is home to approximately 50,000 refugees and is the largest refugee camp on the border. The main health provider in Maela camp is Première Urgence – Aide Médicale Internationale. There are more than 1,200 deliveries in the camp each year and 90% of pregnant women attend the SMRU clinic for antenatal care. The SMRU clinic has a delivery room staffed by locally trained skilled birth attendants and an inpatient and outpatient department staffed by locally trained medics and nurses. An expatriate team consisting of an obstetrician and paediatrician supervise clinical activities and training.

Pregnant women are seen regularly throughout pregnancy. An ultrasound scan is performed in early pregnancy to determine gestation [13]. All women are provided with anaemia prophylaxis, vitamin B1 and tetanus immunisation [10]. If the woman becomes unwell she can present to the clinic at any time where she is assessed and treated by a local skilled birth attendant and medic or doctor if required. Women are encouraged to deliver at the SMRU clinic.

### Definitions

An abortion was defined as a pregnancy ending spontaneously before 28 weeks completed weeks of gestation. A live birth was defined as the delivery of a live born foetus of 28 weeks or more gestation (based on ultrasound) and included multiple births [14]. The 28-week estimated gestational age, rather than the current WHO 22-week cut-off for estimated gestational age was chosen, because no infant respiratory support was available in the clinics. Infants born before 28 weeks who were breathing were given palliative care and excluded from this analysis. An early premature infant was defined as ≥ 28 weeks gestation but < 34 weeks gestation and a late premature infant as 34 to < 37 weeks gestation. Neonatal mortality was defined as the number of neonatal deaths of a live born infant within 28 days of life per 1,000 live births. The number of live births were recorded for all women following antenatal care. Infants were reviewed at 28 days of age and the number of deaths documented. The case notes for all neonatal deaths were examined (by CT) and a single, most probable, cause of death assigned.

### Neonatal services

#### The situation in 2007

In 2007, neonatal care was managed by skilled birth attendants. Due to limited staffing antibiotics were give preferentially by the intramuscular route. Nasogastric feeding was performed while intravenous fluids were rarely used. Care for early premature newborns added significantly to the workload of the skilled birth attendants. The attitude of staff and parents to early premature newborns was fatalistic. Care depended upon parental willingness to help the newborn survive. Without the parents support the birth attendants were unable to provide the care required. The need for development of neonatal services was identified.

#### Development of neonatal guidelines

Locally appropriate standardised neonatal guidelines were developed. Guidelines were based on the WHO integrated management of childhood illness, relevant published literature and expert opinion where evidence-based guidelines were not available. The guidelines were peer reviewed within SMRU, by expatriate and local staff, to ensure they were accurate and appropriate. They were updated and revised annually.

#### Staff training

Staffing was reorganized so that a dedicated team was available to care for admitted newborns. Staff training was introduced in six sequential steps: recognition of the sick neonate, resuscitation of the neonate, examination of a neonate including routine newborn examination, supportive medical care, specific medical care and of counselling parents with premature or sick newborns. The mnemonic MACHO (Milk, Antibiotics, Cord care, Heat and Oxygen) was devised in order to structure the supportive care of the neonate. Training was repeated on a yearly basis. Standardised clinical charts were created for neonatal care and all SCBU admissions were routinely recorded from 2008.

#### Equipping and supplying a dedicated area for neonates

A dedicated area was built to care for unwell neonates and named the special care baby unit (SCBU). The SCBU utilized some existing equipment including resuscitation equipment (self-inflating bag and mask and oral airways), thermometers (glass mercury), stethoscopes and a blood glucose meter (Accu-Chek Aviva, Roche) and additional items were purchased: oxygen cylinders and regulators (0-15l and 0-500ml), an oxygen saturation machine (Hand –held pulse oximeter, model 512, Respironics), suction machine, and apnoea monitors (Breathing monitor, Nanny). In late 2008 jaundice was identified as being a common clinical problem and a serum bilirubin machine (Bilimeter 2, Pfaff Medical) and phototherapy units (home built initially using fluorescent tubing and then, when available, Phillips TL20W bulbs of 315–400 nm wavelength) were obtained.

Essential drugs (antimicrobials (ampicillin, gentamicin, cefotaxime and cloxacillin), anaemia prophylaxis (ferrous sulphate, folate and multivitamins), paracetamol and domperidone) as well as nasogastric tube feeding and intravenous fluids were already available at the SMRU clinic. The neonatal guidelines provided exact information on drug dosing and IV fluid administration.

#### Laboratory support

The field site laboratory was able to provide simple tests including, haematocrit, microscopic urine examination and the fluorescent spot test (a 5 microlitre heel prick sample to measure the activity of G6PD [15]). This qualitative test has been used at SMRU for the last five years and it allows for determination of subjects with G6PD deficiency within 30 minutes of sampling. Electricity for storage of reagents and UV visualization was required. The test can be reliably performed in basic laboratory settings by trained technicians and an external quality control can be performed on the blood spots within few days. Diagnostic microbiology and haematology support was provided by the SMRU laboratories in Mae Sot. 

### Interviews of staff

There was no formal measurement of staff attitude towards neonatal care. Informal interviews were therefore conducted in an effort to capture how this may have evolved over time. Purposive sampling of staff with a good level of English and who had worked with SMRU for a long period were selected as they could offer insights about the outcomes before and after SCBU was established. None of the staff approached to participate declined. The two most senior of seven SCBU medics, one midwife (19 years at SMRU) and the logistician (15 years at SMRU) who supported the development of the SCBU were included. Interviews took place in one day in Maela camp and lasted 15-30 minutes. The interviews were conducted in a private area near the clinic in April 2013. These interviews were transcribed during the course of the interview, typed up and within the next few days the staff were asked to read the transcribed document to verify accuracy. Consent was obtained in writing and participation was voluntary. Responses were de-identified to ensure confidentiality.

### Estimated cost of neonatal care

As care for preterm infants was deemed to be the most expensive, costs for the admission and treatment of these infants were estimated. Calculations were based on the infant requiring a two month hospital admission and receiving four weeks nasogastric tube feeding, five days of intravenous antibiotics, one week of oral antibiotics (amoxicillin and cloxacillin), anaemia prophylaxis, one week of nasal cannula oxygen and supportive treatment (paracetamol and domperidone). Included in this estimation were laboratory costs (blood culture, cerebrospinal fluid culture, full blood count and C-reactive protein (CRP)). Equipment costs were not included in this estimation as they would be dependent on the number of infants admitted to the SCBU and the life span of the equipment.

### Ethical approval

Approval for retrospective analysis of hospital records was granted by the Oxford Tropical Research Ethics Committee (Reference 28-09). OXTREC approval did not specifically waive the consent of the patients to store their data for future use because this is not current practise in any refugee situation anywhere in the world. Retrospective consent was not possible as all records were anonymised.

For the informal staff questionnaire consent was not deemed necessary by the Tak Border Committee Advisory Board because no-risk was thought to be attached [16].

### Statistical analysis

Anonymised data were entered into an Access 2003 database (Microsoft, Redmond WA, USA) and statistical analyses were carried out using Stata 12.1 (StataCorp, College Station TX, USA). Continuous variables were described by the mean or median, where appropriate, and the range given. Logistic regression was used to calculate odds ratios (OR). Chi-squared for trend was used to compare proportions and the Kruskal-Wallis and Wilcoxon rank sum tests were used to compare non-normally distributed variables, with two-tailed P-values of <0.05 indicating significance. 

## Results

### Characteristics of births in Maela camp

Between1^st^ January 2008 and 31^st^ December 2011 there were 5,799 births in Maela camp of which 44 were multiple births (43 sets of twins and 1 set of triplets). There were 5,732 live births. Overall characteristics are presented in Table 1. During this time an increasing proportion of births from mothers who followed antenatal care took place in SMRU with skilled birth attendants, rather than at home with traditional birth attendants (p<0.001) (Table 1).

**Table 1:** General characteristics of MLA deliveries 2008 to 2011.

	**2008**	**2009**	**2010**	**2011**
**All deliveries**	1528	1546	1400	1325
Singletons (% all deliveries)	1502 (98.3)	1517 (98.1)	1374 (98.1)	1317 (99.4)
Multiple deliveries (% all deliveries)	26 (1.7)	29 (1.9)	26 (1.9)	8 (0.6)
Still births (% all deliveries)	15 (1.0)	15 (1.0)	13 (0.9)	19 (1.4)
**Live births**	1513	1531	1387	1306
Male (% live births)	780 (51.6)	793 (51.8)	697 (50.3)	673 (51.5)
Female(% live births)	733 (48.4)	736 (48.1)	690 (49.7)	633 (48.5)
Unknown sex(% live births)	0	2 (0.1)	0	0
**Place of birth**				
SMRU(% live births)	1096 (72.4)	1133 (74.0)	1021 (73.6)	1071 (82.0)
Home(% live births)	304 (20.1)	282 (18.4)	257 (18.5)	133 (10.2)
Hospital(% live births)	113 (7.5)	106 (6.9)	100 (7.2)	95 (7.3)
Other(% live births)	0	10 (0.7)	9 (0.6)	7 (0.5)
**Gestational category**				
Term(% live births)	1398 (92.4)	1408 (92.0)	1262 (91.0)	1215 (93.0)
Late premature(% live births)	86 (5.7)	83 (5.4)	89 (6.4)	73 (5.6)
Early premature(% live births)	29 (1.9)	40 (2.6)	36 (2.6)	18 (1.4)
**Mean EGA in weeks (min, max)**				
Singletons (min,max)	39.1+1.7 (28.5,43.1)	38.9+1.8 (28.0,42.6)	38.8+1.7 (28.1,42.5)	38.9+1.5 (28.2,42.6)
Multiple delivery (min,max)	36.6+2.4 (30.2,39.3)	36.2+3.6 (29.6,41.0)	36.8+2.3 (32.4,40.2)	37.3+0.8 (36.3,38.1)
**Mean weight in g (live births weighed on date of birth)**				
Singletons (min,max)	2927+459 (790,4590)	2948+458 (740,5230)	2981+482 (910,5360)	2991+450 (1300,4570)
Multiple delivery (min,max)	2193+416 (1510,2940)	2230+501 (1230,3080)	2313+348 (1790,2810)	2424+345 (1740,2820)
**Median Apgar score 1 minute (SMRU births)**				
Singletons (min,max)	8 (3,9)	8 (1,9)	9 (2,9)	9 (1,10)
Multiple delivery (min,max)	8 (6,8)	8 (4,9)	8 (6,9)	9 (8,9)
**Median Apgar score 5 minutes (SMRU births)**				
Singletons (min,max)	9 (5,10)	9 (1,10)	10 (3,10)	10 (2,10)
Multiple delivery (min,max)	9 (8,9)	9 (7,10)	9 (8,10)	10 (9,10)
**Neonatal deaths**	33	22	17	14

### Admission characteristics of SCBU neonates

There were 970 admissions of 952 infants to the SCBU over 4 years, 97.9% (934) of these mothers followed ANC in Maela camp. There were 11 admissions of infants less than 28 weeks for palliative care of whom 10 died and 1 was alive at one year of age. The following analysis takes into account only those infants ≥ 28weeks and those infants born to mothers following ANC in Maela camp (n=923).

The number of admissions increased over time, from 12.0% (181/1513) of all live births in 2008 to 19.8% (215/1306) in 2011. More male infants were admitted than female and the majority of admissions were of term (≥ 37 weeks) gestation (Table 2). The birth weight of admitted infants increased over time. Of all infants admitted to SCBU, 29.7% (274/923) were born less than 37 completed weeks. Although there was no change in the number of late preterm infants admitted to the SCBU there were less early preterm (Table 2).

**Table 2:** Characteristics of infants admitted to SCBU.

	**2008**	**2009**	**2010**	**2011**
**Admissions**				
Total number (% live births)	181 (12.0)	215 (14.0)	268 (19.3)	259 (19.8)
Median age at admission days (range)	1 (0 - 42)	1 (0 - 25)	1 (0 - 24)	2 (0 - 19)
Length of admission days (range)	5 (0 - 42)	5 (0 - 63)	5 (0 - 53)	4 (0 - 44)
Late premature infants (% admissions)	31 (17.1)	35 (16.3)	55 (20.5)	47 (18.1)
Early premature (% admissions)	26 (13.6)	37 (17.0)	26 (9.6)	17 (6.4)
**Deaths in SCBU**				
Total (% of admissions)	22 (12.2)	10 (4.7)	12 (4.5)	8 (3.1)
Late premature infants (% late preterm admissions)	3 (9.7)	0	2 (3.6)	0
Early premature (% early preterm admissions)	11 (42.3)	7 (18.9)	8 (30.8)	3 (17.6)

### Diagnosis and treatment of infants admitted to SCBU

**Figure 1:**
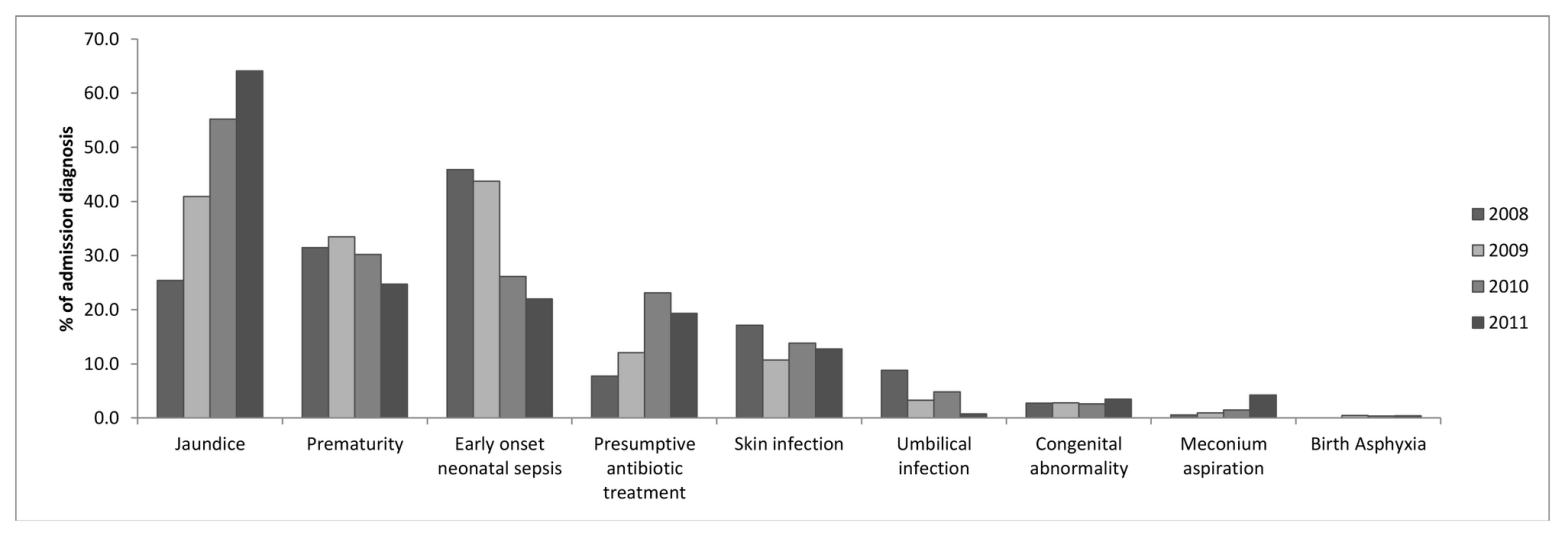
Diagnosis of infants admitted to SCBU. Infants could have more than one diagnosis on admission.

The most common reason for admission to SCBU changed over the years. Early onset neonatal sepsis (less than seven days of age, EONS) was the commonest admission diagnosis in 2008, but by 2011 the treatment of jaundice had become the most common reason for admission (Figure 1). Correspondingly, the proportion of infants who received phototherapy increased from 25.4% (46/181) to 64.1% (166/259) of all admissions in 2011 (p <0.001). Of the early preterm infants, 68.6% (72/105) received supplemental oxygen during their admission compared with 14.2% (91/641) of term infants. However in absolute numbers, more term infants received oxygen than early preterm infants (91 compared to 72) over the four year period. The proportion of infants admitted to SCBU who received intravenous therapy and oxygen therapy significantly decreased from 2008 until 2011 (p=0.006 and p=0.04 respectively) (Table 3).

**Table 3:** Summary of number of infants who received intravenous treatment, oxygen and phototherapy.

	**2008**	**2009**	**2010**	**2011**
	**(% of admissions)**	**(% of admissions)**	**(% of admissions)**	**(% of admissions)**
IV fluid / drugs	112 (61.9)	138 (64.2)	147 (54.9)	121 (46.7)
Oxygen	49 (27.1)	49 (22.8)	55 (20.9)	43 (20.5)
Phototherapy	46 (25.4)	88 (40.9)	148 (55.2)	166 (64.1)

### Mortality

**Figure 2:**
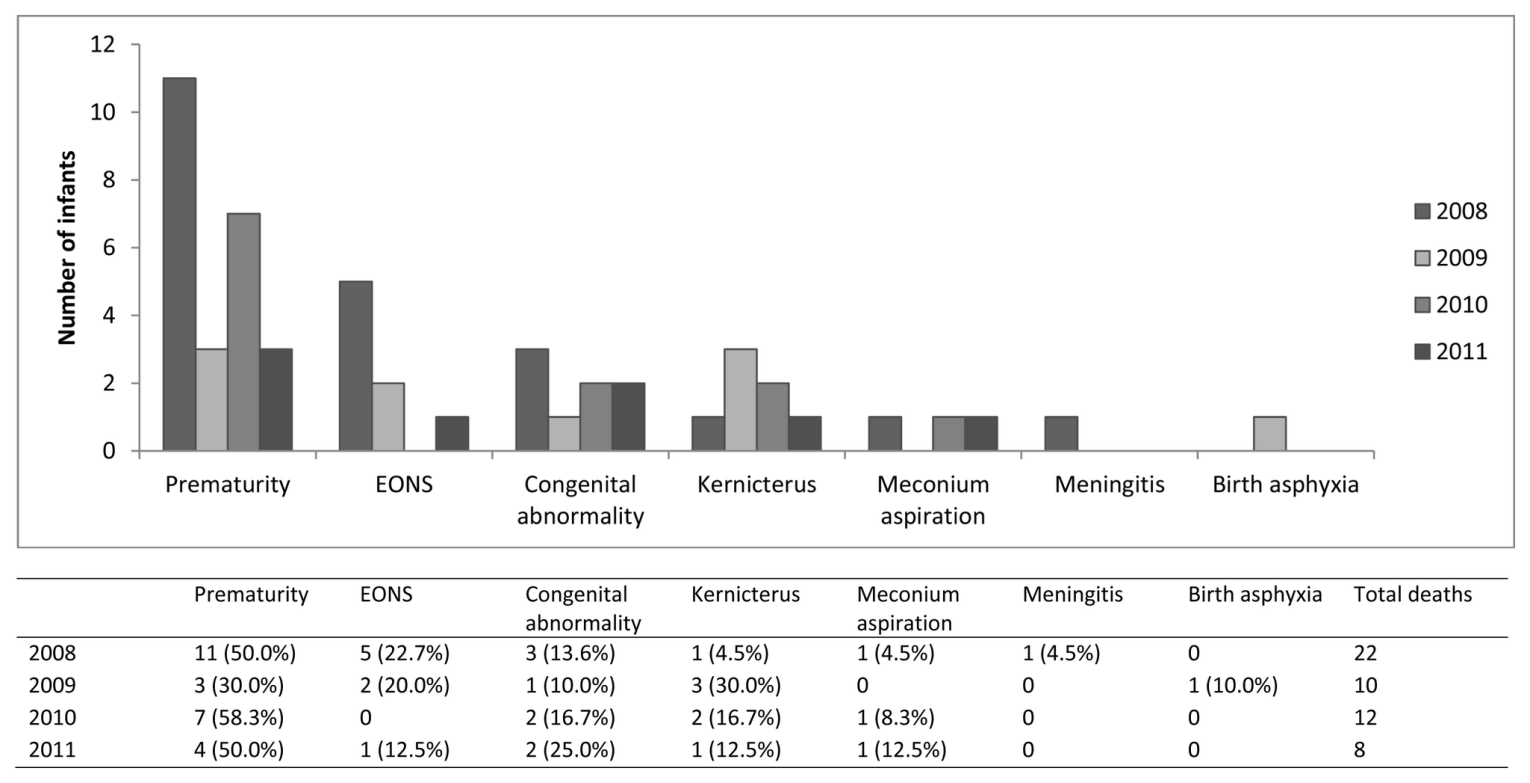
The main cause of death of infants addmited to SCBU.

The proportion of infants who died following admission to SCBU fell from 12.2% (22/181) in 2008 to 3.1% (8/259) in 2011 (p<0.001). This is probably explained in part by the increasing number of babies admitted for jaundice. Cause specific mortality fell in all of the four main causes of death overall (prematurity, EONS, congenital abnormality and jaundice) (Table 4). Prematurity remained the commonest cause of death over the four years reported here despite a significant fall in mortality from 19.3% (11/57) in 2008 to 4.8% (3/63) in 2011 (p=0.03) (Figure 2). Over the entire time period reported here, early premature infants were at a significantly greater risk of death than term and late preterm infants (OR 9.5 95% CI 5.4-16.5, p<0.001). The risk of late preterm infants dying was not significantly different compared to term infants (OR 1.4 95%CI 0.8-2.6, p=0.3).

**Table 4:** Cause specific mortality from 2008 to 2011.

**Cause of death**	**2008**	**2009**	**2010**	**2011**
	**(%)**	**(%)**	**(%)**	**(%)**
Prematurity	11/57 (19.3)	3/72 (4.2)	7/81 (8.6)	3/63 (4.8)
Early onset neonatal sepsis	5/83 (6.0)	2/94 (2.1)	0/70	1/57 (1.8)
Jaundice	1/46 (2.2)	3/88 (3.4)	2/148 (1.4)	1/166 (0.6)
Congenital abnormality	3/5 (60.0)	1/6 (16.7)	2/7 (28.6)	2/9 (22.2)

**Figure 3:**
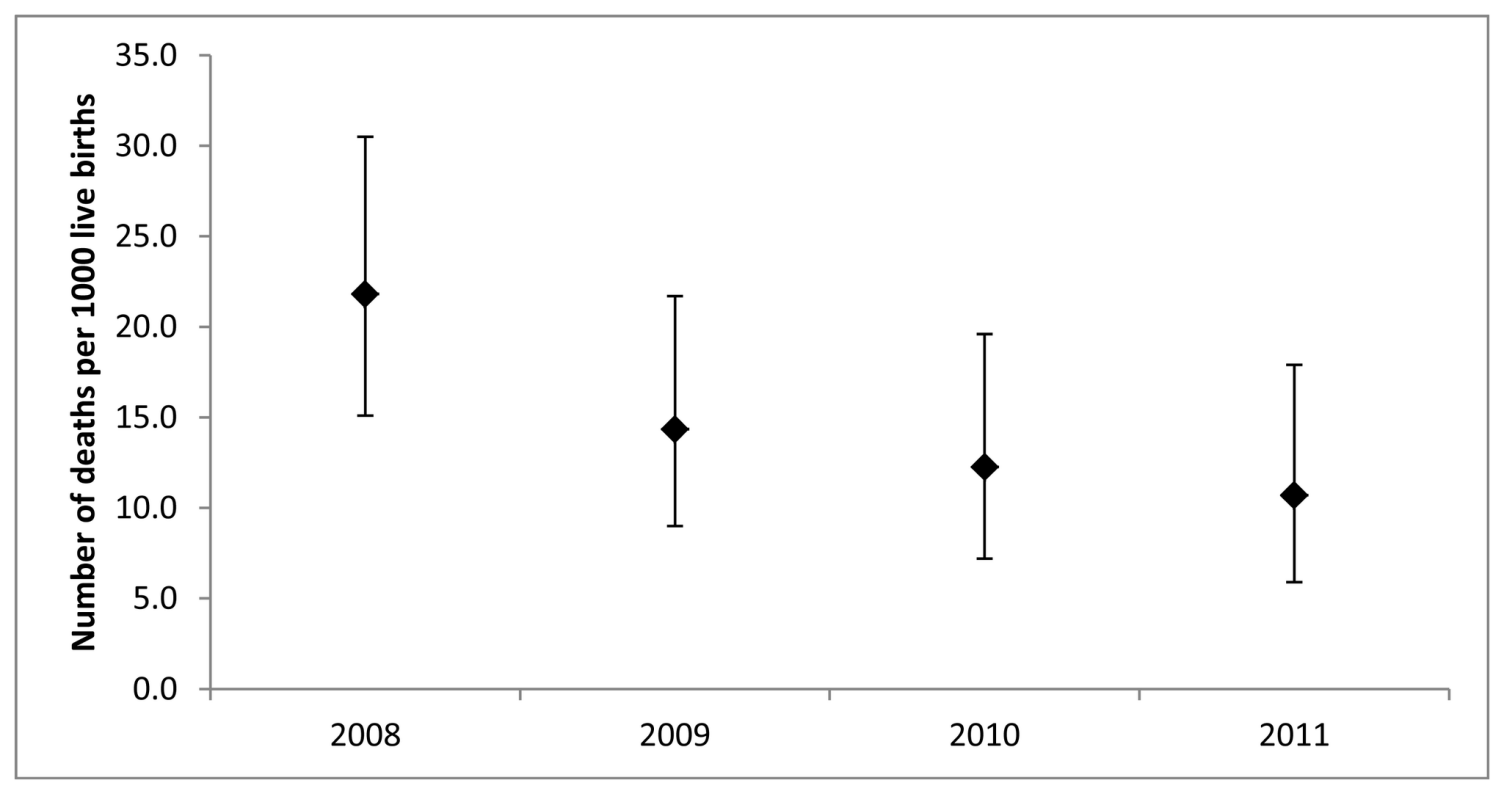
Neonatal mortality in Maela Camp.

From 2008 until 2011 neonatal mortality within Maela camp significantly decreased from 21.8 deaths per 1000 live births to 10.7 deaths per 1000 live births (p =0.03) (Figure 3).

### Neonatal sepsis and presumptive antibiotic administration

The proportion of infants admitted to the SBCU with a clinical diagnosis of EONS decreased from 85/181 (45.7%) in 2008 to 58/259 (22.1%) in 2011 (p<0.001), however the incidence of EONS in the population did not change (56.2 per 1000 live births in 2008 compared with 44.4 per 1000 live births in 2011, p=0.2). Infants admitted to SCBU for presumptive antibiotic treatment, i.e. those infants with risk factors for EONS but not displaying the full clinical syndrome, rose from 14/181 (7.5%) in 2008 to 50/259 (19.0%) in 2011 (p<0.001).

Four hundred and twenty four infants (45.5% of all) admitted to SCBU had blood taken for bacterial culture, with a median blood volume cultured of 0.8 ml (Interquartile range 0.4-1.1). Seven (1.6%) cultures grew a significant bacterial isolate: three *Escherichia coli* (3/7, 43%) and one isolate each of *Enterobacter cloacae*, Group B Streptococcus, *Klebsiella pneumoniae* and *Staphylococcus aureus.*

### Jaundice

Blue light phototherapy was introduced in August 2009. To establish phototherapy usage, and which conditions might require longer treatment, data were recorded to determine the exact length of phototherapy treatment and the possible cause of jaundice between August 2009 and December 2010. The possible causes that could be identified included prematurity, G6PD deficiency, sepsis, polycythaemia (capillary haematocrit >70%) and a weight loss of >10%. An infant could be given more than one diagnosis and infants in whom a diagnosis was not identified were assigned to the unknown group.

The median age at starting phototherapy was 82 hours (range 9-310 hours) and the median length of treatment was 27 hours (range 4-146 hours). The commonest possible cause of jaundice was prematurity (38.1% (43/113)). G6PD deficiency was diagnosed in 28/113 (24.8%) of infants and approximately one third of infants (35/113) had no identifiable cause of jaundice. Infants with G6PD deficiency needed significantly longer phototherapy treatment than those who had normal G6PD activity (a median of 49 hours compared with 24 hours, p=0.001). Over this time period eight infants who received phototherapy died from kernicterus and four of these were G6PD deficient.

Routine newborn screening for G6PD deficiency started in January 2011. From 1st January 2011 until 2nd March 2012 1,571 infants were tested. One hundred and thirteen (7.2%) infants were found to be deficient and there was a large ethnic variation. Sgaw Karen infants were found to have the highest incidence of G6PD deficiency with 81/483 (16.8%) of males and 14/521 (2.7%) of females tested being deficient. Of all of the G6PD deficient infants, 54/113 (47.8%) needed admission to SCBU for the treatment of jaundice.

### Interviews with staff

Staff were able to identify different components of SCBU care and spoke freely of how they used them in their daily work. Two major themes emerged from the transcripts of interviews including: 1) We know what to do and 2) SCBU is good. The medics expressed confidence in their training and their ability to recognize and respond to problems in neonates. All of the staff interviewed felt that the additional staffing and equipment in SCBU meant that more neonates, especially premature infants had an increased chance of survival. They also reported on positive comments that had been given, by families of infants treated in SCBU, saying that it was understood in the population that treatment in SCBU meant more chance of survival for the infant (file S1).

### Cost

The total cost of the admission of a premature infant requiring a two month hospital stay was calculated as being $435.94 or $7.27 per day (file S2). However this figure does not take into account the expatriate paediatricians salary. It is difficult to compare these costs directly to developed countries because of global differences in staffing costs.

## Discussion

In this manuscript we have shown that it is possible to provide effective low cost neonatal care. We have presented a framework of capacity building for high quality neonatal care in a resource poor setting. In doing so the proportion of infants who survived to be discharged home from SCBU increased over time including premature infants. Mortality from prematurity, EONS, congenital abnormality and jaundice decreased over the time period described here. In addition, on a population level, the number of neonatal deaths halved in Maela camp from 2008 until 2011. One can conclude that the development and implementation of the SCBU was a factor in this achievement since other relevant influences, such as antenatal care remained stable apart from a reduction in homebirths.

SMRU was a unique setting in which to assess neonatal interventions because of its track record of providing intensive antenatal care and a high level of deliveries with skilled birth attendants. As previously stated effective neonatal care begins with the care of the pregnant women and this was already in place at SMRU. Therefore, any additional benefit seen in the neonatal population were most likely to have been due to interventions occurring after birth.

The first step in the training of local health care workers was the recognition of a sick neonate. The effectiveness of this training was demonstrated by the fact that, over time, the number of infants who were admitted to the SCBU for presumptive antibiotic treatment increased. This implies that skilled birth attendants, nurses and medics were identifying infants at risk of neonatal sepsis earlier, and before the full clinical syndrome of neonatal sepsis was revealed. Over the same time period the number of infants dying from neonatal sepsis decreased. Early recognition was therefore potentially saving lives.

Approximately one third of all the admissions to the SCBU were for prematurity. Preterm births account for three quarters of all perinatal mortality globally and survivors are at risk of developing serious long term sequelae [14]. In our setting infants less than 28 completed weeks of gestation were not considered viable and hence did not appear in the outcome statistics. However, any infant born less than 28 weeks but showing signs of life received palliative care in the unit. These clear cut gestational definitions could be used since the majority of pregnant women delivering at the SMRU clinic had an accurate dating ultrasound in early pregnancy.

There was no provision of ventilatory support in the SCBU clinic because this was beyond the level at which the unit could operate, given staff skill and funding limitations. The aim therefore was to concentrate on proven simple interventions [17]. All preterm infants received supportive care using the mnemonic MACHO as a guide, in order to provide simple interventions at the highest possible standard. These were shown to be effective with a significant reduction in the proportion of preterm infants dying in the SCBU over the time period studied.

The number of infants admitted to SCBU for treatment of jaundice increased dramatically over the four years reported. One important reason was the staff recognition of infants who were jaundiced and this recognition may have increased with familiarity of the condition. In 2009 when the serum bilirubin measurement was introduced, an objective result could be determined and treatment initiated accordingly.

The cause of jaundice in these infants is interesting. Maela camp is predominately inhabited by refugees of Sgaw Karen origin. This population has a high incidence of G6PD deficiency [18]. From the observed data, it is apparent that G6PD deficiency plays a part in the cause of neonatal jaundice in this population. Half of the infants with G6PD deficiency required treatment for their jaundice. These infants also required longer phototherapy treatment than infants without G6PD deficiency. Of the deaths reported from kernicterus, half of the infants had G6PD deficiency. The exact mechanism by which G6PD might play in causing jaundice is unknown but thought not to be directly related to haemolysis [19]. Of the infants treated between August 2009 and December 2010, the cause of jaundice was unknown in 30% of infants. These infants needed significantly longer treatment with phototherapy compared with infants who did have a diagnosis. This implies a more serious illness and confirms that further work on the cause of jaundice is a priority for this population.

Staff reported that with teaching and training they learned how to take care of neonates. They liked and followed the protocols and expressed confidence in their ability to respond to problems in neonates. They thought that the early premature neonate had a better chance with SCBU than in the past when there was no SCBU. Positive impressions of how the people in the camp now responded to premature and sick infants were reported.

Caring for preterm infants in an intensive care setting is expensive. In 2000 it was estimated that the costs associated with neonatal care amounted to $10.2 billion in the USA [20]. The cost of providing care to a preterm infant while in the SCBU at SMRU was approximately $7 a day. This estimate did not include over heads or daily running costs of the unit (such as electricity, logistics etc.) as the aim was to identify the additional costs to SMRU, a unit with an existing infrastructure and support team. Experience and expertise is needed to establish such a unit, in this case a British consultant paediatrician. Therefore if a unit was to be developed de novo, these costs would have to be taken into consideration along with staff costs (local and expatriate), which might not be comparable to those in other geographic locations, especially when antenatal and delivery care were lacking.

There are limitations to the observations reported here, the predominant one being that this is a retrospective descriptive study so cautious interpretation of significant P-values is required as other factors not identified may have contributed to the observed changes. There was a reduction in the proportion of home deliveries over the time period reported. This also could have been a factor in the reduction of neonatal mortality. All infants born in the unit were medically assessed <24 hours after birth. Therefore potential medical problems were identified and treated early. In addition, in 2011 there was a decrease in the number of multiple births; this may also have contributed to the reported decreased mortality. On-going work will see if this trend continues and examine its possible impact on the reduction of neonatal mortality.

It would have been interesting to have a formal assessment of staff knowledge, attitude and confidence towards neonatal care measured before, during and at the end of the period of the data reported in this manuscript. While neonatal care increases survival of preterm infants they are prone to a range of long term complications compared to term infants and there is a paucity of data on developmental outcomes from resource limited settings [21]. Data on neurological developmental takes time to accumulate as many important developmental outcomes are not obvious until the child reaches an age where he or she can walk and talk, they have not been reported here as the follow up is currently on going.

## Conclusion

We have shown that it is possible to develop high standard, effective neonatal care in a financially constrained setting run by local health workers. In addition we postulate that that this approach contributed to the halving of neonatal mortality in the population that it served over a four year time period.

## Supporting Information

File S1 **Results of staff interviews regarding attitudes towards neonatal care in Maela camp.**
(DOCX)

File S2 **Cost calculation for care of a premature infant SMRU SCBU.**
(DOCX)
