# Supplementary material for: Neonatal Intensive Care in a Karen Refugee Camp: A 4 Year Descriptive Study
Source: PLoS One. 2013 Aug 22;8(8):e72721. doi: 10.1371/journal.pone.0072721 (PMC3749980; doi:10.1371/journal.pone.0072721)
Supplement: File S1 — Results of staff interviews regarding attitudes towards neonatal care in Maela camp. (DOCX) [file pone.0072721.s001.docx]

## Supplemental material S1: Results of staff interviews regarding attitudes towards neonatal care in Maela camp

The following open-ended questions were asked:

1. Have you ever worked in a SCBU before?
2. Can you tell me about your experience of SCBU in Maela?
3. How do you feel about the survival for a 30 week newborn before and after we had SCBU?
4. What do you think or did you hear from the people about SCBU?
5. Do you have any example of feedback from them about neonatal care?

Transcript of interview with medical and logistical staff working in the SMRU clinic Maela Camp

*We know what to do*:

*“For me I already work as a nurse for a long time in Burma in children less than 12 years old and when I am arrive in SCBU I have more learning from the doctor and how to take care of small babies, and more experience and knowledge.* (Medic 1)

*“We go smoothly with the protocol”* (Medic 1)

*“Before I did not work in ICU or in SCBU but I take care the small baby. With SCBU we know how to examine the neonate in the proper way, we know diseases especially for the neonate, we know about the EGA (estimated gestational age), the difference between the premature and the term, after doctor we know more specifically the management for neonate.”* (Medic 2)

“*If we have apnoea we exactly know how to take care quickly to make the baby breath again*” (Medic 1)

*“Before we are scared and now we have more confidence, not 100% but more”.* (Medic 2)

*SCBU is good:*

*“I think we feel different before SCBU; we worry too much for 30 week baby, we have not use incubator, we have staff not enough and also oxygen not enough. The SCBU has special staff to take care the baby, the protocol is very good, the area for the neonate is better and I think in the mind of the parents they feel this is helpful for them. This is better than before (SCBU)”* (Midwife).

*“We admit the premature ones for a long time, maybe one or one and a half months and the parents tell us when they are discharge(d) they are very happy because they know the baby is too small”* (Medic 2).

*“In the SCBU the medic, the whole team and the mother we work together and this is better for baby alive”* (Medic 1).

*“Before this we feel not very comfortable to take the 30 weeks and now we feel better because the SCBU is successful for most of the baby and the mother is happy”* (Midwife).

*“With the SCBU we are more hopeful for 30 week baby. Very premature we know is difficult but we know we can explain to the parents when we admit them. Parents too know their baby more chance to survive in the incubator and the other special treatment for them”* (Medic 2).

*“We saw the problem with the small baby and we know they cannot survive and we cannot solve this problem. Now we know that the people if they have the small baby or the sick baby in the camp they will come to the SCBU for the treatment and the care. Now we see it improve for these babies and stay alive”* (Logistic).
