# Supplementary material for: Neonatal Intensive Care in a Karen Refugee Camp: A 4 Year Descriptive Study
Source: PLoS One. 2013 Aug 22;8(8):e72721. doi: 10.1371/journal.pone.0072721 (PMC3749980; doi:10.1371/journal.pone.0072721)
Supplement: File S2 — Cost calculation for care of a premature infant SMRU SCBU. (DOCX) [file pone.0072721.s002.docx]

## Supplemental material S2: Cost calculation for care of a premature infant SMRU SCBU

Cost estimated for a preterm infant requiring a two month hospital admission and receiving four weeks nasogastric tube feeding, five days of intravenous antibiotics (ampicillin and gentamicin), one week of oral antibiotics (amoxicillin and cloxacillin), anaemia prophylaxis, one week of nasal cannula oxygen and supportive treatment (paracetamol and domperidone)

|  | **Number** | **Item cost (THB)** | **Total cost ($)** |
| --- | --- | --- | --- |
| **Medical supplies** |  |  |  |
| Nasogastric tube | 5 | 10.7 | 1.78 |
| 20 ml syringe | 5 | 5.6 | 0.93 |
| 5 ml syringe | 40 | 1.8 | 2.40 |
| IV cannula | 4 | 16.1 | 2.15 |
| Nasal oxygen cannula | 4 | 60.8 | 8.11 |
| **Medication** |  |  |  |
| Normal saline 500ml bottle | 7 | 37.0 | 8.63 |
| 10% dextrose 500ml bottle | 7 | 50.0 | 11.67 |
| 50% dextrose | 7 | 8.0 | 1.87 |
| Ampicillin | 14 | 18.0 | 8.40 |
| Gentamycin | 5 | 6.0 | 1.00 |
| Cloxacillin | 1 | 16.1 | 0.54 |
| Amoxicillin | 1 | 13.9 | 0.46 |
| Paracetamol | 1 | 4.2 | 0.14 |
| Domperidone | 1 | 15.0 | 0.50 |
| Multivitamins | 12 | 25.0 | 10.00 |
| Folate | 10 | 0.1 | 0.03 |
| Oxygen | 1 | 500.0 | 16.67 |
| **Investigations** |  |  |  |
| Blood culture | 1 | 250.0 | 8.33 |
| CSF culture | 1 | 100.0 | 3.33 |
| Complete blood count | 1 | 38.0 | 1.27 |
| C-reactive protein | 1 | 112.0 | 3.73 |
| Haematocrit tubes (one bottle) | 1 | 70.0 | 2.33 |
| ****Staff (cost per month)** |  | |  |
| Medic |  | 5000.0 | 166.67 |
| Nurse |  | 3000.0 | 175.00 |
| **Total for two months admission** | | | **435.94** |
| **Total per day** | | | **7.27** |

* based on an exchange rate on $1 = 30 THB

** based on three shifts per day; one medic per shift and two nurses per shift, (one medic and nurse on annual leave) caring for 8 infants
